# Effects of mobile application interventions on quality of life in patients with cancer: Protocol for a systematic review and meta-analysis

**DOI:** 10.1371/journal.pone.0314590

**Published:** 2025-02-12

**Authors:** Yuying Zhang, Yuhan Zhang, Wansheng Li, Xiaoya Hou, Peili Zhang

**Affiliations:** 1 Academy of Medical Sciences, Shanxi Medical University, Taiyuan, China; 2 School of Nursing, Shanxi Medical University, Taiyuan, China; 3 First Hospital of Shanxi Medical University, Taiyuan, China; National Defense Medical Center, TAIWAN

## Abstract

**Background:**

Cancer is a public health burden that seriously affects patients’ daily quality of life. Mobile applications are increasingly being used in the field of health promotion, but the intervention effect of mobile apps on the quality of life of cancer patients is still inconsistent.

**Methods:**

We will use key words and medical subject headings to search for randomized controlled trials in nine databases until January 10, 2024. The quality of the studies will be assessed using the risk of bias tool recommended in the Cochrane risk-of-bias tool, and a pooled analysis of data will be conducted using random effects models to assess the impact of mobile application interventions on pain and quality of life in cancer patients.

**Results:**

We will publish our findings in a peer-reviewed journal.

**Conclusion:**

We will draw conclusions based on the outcomes of the systematic review and meta-analysis.

**Prospero registration number:**

CRD42024497649.

## Introduction

In recent years, the incidence and mortality rates of malignant tumors have increased worldwide, and the number of patients has risen dramatically. In 2020, an estimated 19.3 million new cases of cancer and 10 million cancer-related deaths occurred globally, of which 4.57 million new cases and 3 million deaths occurred in China, both ranking first in the world, according to the WHO’s International Agency for Research on Cancer [1]. In terms of reduced quality of life, cancer imposes a heavy burden on individuals and society [2]. Patients may face physical deterioration that directly affects their ability to carry out daily activities, making simple activities such as walking, eating, sleeping, etc. extremely difficult, leading to social isolation and increased loneliness. In addition, cancer patients require long-term medical care and nursing, which not only raises the consumption of medical resources but also places greater demands and challenges on medical institutions and social medical security systems. With the advancement of medical technology, the survival rate of cancer is constantly improving [3], but the illness still significantly decreases their quality of life and may have a negative effect on their course of therapy. For example, a number of studies indicated that between 75 and 99% of cancer patients had fatigue, which affects their daily lives and their chances of survival [4]. Therefore, effective interventions for cancer patients are desperately needed in order to assist them in enhancing their overall quality of life and relieving pain.

As telemedicine expands globally, mobile applications are contributing significantly to promoting the health of cancer patients and have become an encouraging tool in managing the disease process and improving their physical function. It is a stand-alone piece of software that resides on mobile devices to monitor patients, collect objective data, help patients get better, improve patient satisfaction, and provide resources to patients and their healthcare providers [5]. It can also enable physician-to-physician, physician-to-patient, and patient-to-patient relationships regardless of location, provide online guidance to patients, improve patients’ health awareness and compliance, and meet patients’ health needs at different levels [6]. Mobile apps can improve cancer patients’ quality of life in many ways, not just in one area such as symptom management, pain control, social support or self-efficacy. They typically integrate a variety of functions, such as health record management, medication reminders, symptom tracking, psychological support, social interaction, nutritional guidance, etc., to provide patients with a one-stop health management service. This completeness and comprehensiveness allow patients to meet multiple needs on one platform, thereby improving their overall quality of life.

So far, mobile applications have focused on diabetes, psychiatry, heart failure, kidney disease and other areas, and research on cancer patients is still relatively limited. And it is unknown how they would affect cancer patients’ quality of life. Çınar et al [7] demonstrated a substantial enhancement in QoL in the mobile app group when compared to the control group by combining symptom diary monitoring with mobile app management and health education for patients with breast cancer. A scoping review published by Aapro et al [8] suggested that digital health solutions offered an innovative approach to oncology support. Di and Li [9] discovered that mobile apps helped reduce the occurrence of oral symptoms and increase QoL in nasopharyngeal cancer. At the same time, Sui et al [10] conducted a WeChat-based education and rehabilitation program for non-small cell lung cancer patients to assist reduce anxiety and despair and improve quality of life. However, Lee et al [11] utilized the ePRO-CTCAE app to evaluate symptom management and quality of life in cancer patients and ultimately did not reveal any differences in QoL changes. A study conducted by Graetz et al [12] showed that providing self-managed app support had no significant impact on health care utilization and QoL for cancer patients. Furthermore, Greer et al [13] found that mobile apps did not enhance drug compliance among cancer patients. Ultimately, we found conflicting literature and insufficient evidence. Therefore, a comprehensive quantitative synthesis of the impact of mobile apps on the quality of life of cancer patients is needed to provide researchers and oncologists with important up-to-date information on the details of life management in cancer.

Although numerous studies [14–16] have investigated the potential advantages of mobile app interventions for cancer patients, they have primarily focused on the timing of intervention, theoretical application, treatment strategies, physical activity and so on. However, there is a scarcity of in-depth reviews of the actual implications of intervention on a particular mobile application. In light of this, the purpose of this study is to fill a gap and further investigate the specific impact of mobile gaming and rehabilitation training applications in order to identify more efficient and appropriate mobile applications. Additionally, the effectiveness of various QoL assessment tools in reflecting patients’ quality of life will be investigated using subgroup analysis, with the goal of guiding clinicians to more accurately match patient characteristics when selecting assessment tools and improving assessment accuracy.

## Methods

### Design

This protocol aimed to investigate the effectiveness of mobile application interventions on quality of life in cancer patients. The study has been registered in the International Prospective Register of Systematic Reviews (PROSPERO) under the registration number CRD42024497649. It will be completed in accordance with the updated Preferred Reporting Items for Systematic Review and Meta-analyses (PRISMA2020) guidelines [17].

### Inclusion and exclusion criteria

#### Inclusion criteria

The individuals will be diagnosed with cancer by pathological histology, regardless of their gender, race, cancer site, kind, and stage, or the type of cancer treatment they had undergone. The intervention will consist of implementing a nursing intervention in the form of an application (APP). The control group will consist of those receiving routine care (e.g. usual care, telephone follow-up, manual instructions, traditional health education without the use of a mobile app, waitlist control, etc.). The outcome of this review is quality of life, which is mainly assessed in specific categories such as physical, emotional, functional, social well-being, symptoms, and so on, utilizing assessment instruments such as the European Organization for Research and Treatment of Cancer Quality-of-Life Questionnaire Core 30 (EORTC QLQ-C30), 36-item Short Form Health Survey (SF-36), the World Health Organization Quality of Life-BREF questionnaire (WHOQOL-BREF), the 27-item Functional Assessment of Cancer Therapy-General (FACT-G), etc. RCTs will be included. And grey literature such as conference proceedings, thesis work and preprints will be included. Only documents written in English or Chinese will be considered.

#### Exclusion criteria

Patients without cancer; research proposals, journal articles, newspaper articles, and conference papers; studies not available in full text, lacking original data, or duplicates; studies using telehealth, websites, SMS text messages, emails, or other technological interventions that did not involve mobile apps; non-randomized controlled trials, animal studies, narrative reviews, editorials and case reports.

### Search strategy

Nine electronic databases, including Cochrane Library, MEDLINE, EMBASE, Web of Science, PubMed, China National Knowledge Infrastructure (CNKI), China Biomedical Literature Service System (SinoMed), Weipu Information Chinese Periodical Service Platform (VIP) and WanFang Data Knowledge Service Platform (WanFang), will be searched from inception to January 10, 2024. In addition, we will also review the references included in the trial to find more relevant studies. Mesh terms and free words will be used in combination to carry out the search strategies. English search phrases will include (“APP” OR “mobile apps” OR “mobile application*” OR “smartphone*”) AND (“cancer*” OR “tumor*” OR “tumour*” OR “malignan*” OR “onco*” OR “neoplas*” OR “carcinoma*”) AND (“quality of life” OR “life quality” OR “health related quality of life” OR “HROOL”) AND (“random*” OR “controlled clinical trial*” OR “single blind” OR “double blind” OR “placebo” OR “RCT”). A manual review of the reference lists of the chosen publications will be conducted to identify other pertinent records. Two investigators (Zhang and Li) will perform the searches independently. EndNote will be used to import and manage selected documents. Please refer to Table 1 for search tactics.

**Table 1:** The Pubmed search strategy.

Database	Search step	Search strategy	Search result
PubMed			
	#1	Mobile Applications[MeSH Terms]	
	#2	(((mobile application*[Title/Abstract]) OR (APP[Title/Abstract])) OR (mobile apps[Title/Abstract])) OR (smartphone*[Title/Abstract])	
	#3	#1 OR #2	
	#4	Neoplasms[MeSH Terms]	
	#5	((((((cancer*[Title/Abstract]) OR (tumor*[Title/Abstract])) OR (tumour*[Title/Abstract])) OR (malignan*[Title/Abstract])) OR (onco*[Title/Abstract])) OR (neoplas*[Title/Abstract])) OR (carcinoma*[Title/Abstract])	
	#6	#4 OR #5	
	#7	Quality of Life[MeSH Terms]	
	#8	(((Life Quality[Title/Abstract]) OR (Health Related Quality of Life[Title/Abstract])) OR (HROOL[Title/Abstract])) OR (Quality of life[Title/Abstract])	
	#9	#7 OR #8	
	#10	(((((random*[Title/Abstract]) OR (controlled clinical trial*[Title/Abstract])) OR (RCT[Title/Abstract])) OR (single blind[Title/Abstract])) OR (double blind[Title/Abstract])) OR (placebo[Title/Abstract])	
	#11	#3 AND #6 AND #9 AND #10	

### Screening and selection

**Fig 1:**
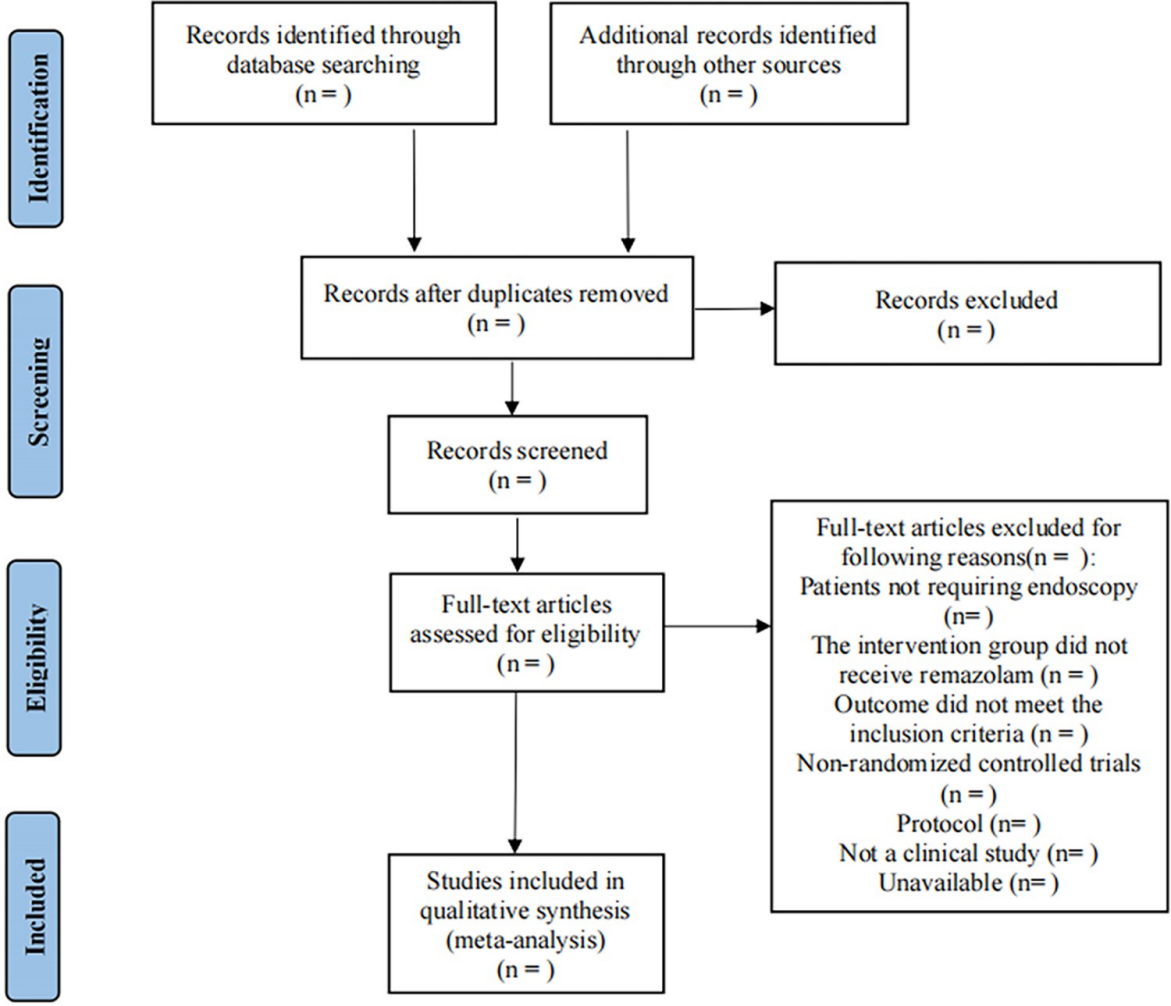
Flow diagram of study selection.

The records will be loaded into EndNote X9 (Clarivate Analytics). Two researchers will utilize EndNote to detect and manually eliminate duplicates. Using the inclusion and exclusion criteria, two authors (Zhang and Li) will independently screen the titles, abstracts and keywords of the remaining publications. Following a preliminary screening, all possibly eligible papers will be downloaded in full, and two authors will independently evaluate each one in light of the eligibility requirements. Any differences of opinion will be worked out through discussion or advice from a third author (Hou). A flowchart of the process used to select trials is shown in Fig 1.

### Data extraction

We will extract data from the selected trials using a pre-designed format (as shown in S1 Table). The details that will be collected are the initial author’s name, date of publication, country, sample size, participant characteristics (mean age, type, stage of cancer, treatment strategy), type of intervention and control, length of intervention, and outcome measures. Two authors (Zhang and Li) will review the extracted data and disagreements will be handled by discussion or by a third author. If some data are not available, we will contact the original author.

### Assessment of risk of bias

This review will be appraised for the methodological quality of all included RCTs according to the Cochrane Handbook for Systematic Reviews of Interventions [18]. The risk of bias assessment encompasses generation of the allocation sequence, allocation concealment, blinding of the participants and personnel, blinding of outcome assessors, selective outcome reporting, inadequate outcome data, and other possible sources of bias. According to the study data, each risk of bias will be categorized as low, unclear, or high. All studies are examined independently by two reviewers (Zhang and Li). If there are any inconsistencies, the two authors will discuss them before turning to the third author (Hou).

### Data synthesis

We are going to use RevMan (version 5.4) and Stata (version 18.0) to assess the heterogeneity. The χ^2^ test and I^2^ statistic will be used for the assessment of heterogeneity between studies. A fixed-effect model will be used for the combination of data if p> .1 and I^2^<50%. If p< .1 and I^2^>50%, a random-effect model will be applied to combine the data, and obvious heterogeneity between the studies is taken into account, and subgroup analysis should be performed according to participants, outcome characteristics, and methodological factors such as cancer type, intervention duration, intervention form, and assessment tools. If it is not possible to determine the source of heterogeneity, we will conduct a descriptive analysis to seek significant components without substantial heterogeneity through the variables used in the subgroup analysis and the outcomes. Relative risk (RR) will be used as the effect size. Continuous variables will be expressed as mean ± standard deviation. The weighted mean difference (WMD) will be used as the effect size for continuous variables when the units are the same, and the standardized mean difference (SMD) when the units are different. The combined effect size of both will be reported as the effect size and its 95% CIs. Sensitivity analysis, if allowed by the data, will be implemented to test the robustness and reliability of the overall analysis. And a funnel plot and Egger’s test will be performed to calculate publication bias using Stata software.

### Meta-biases

To check for reporting bias, we will investigate whether study procedures were released before data collection or patient recruitment. If ten or more studies are present, we will also assess the funnel plot.

### Confidence in cumulative estimate

The two authors (Zhang and Li) will use the Grading of Recommendations Assessment, Development and Evaluation (GRADE) methodology scale [19] to independently evaluate the quality of the evidence, and any disagreements will be determined by discussion or by a third author (Hou). The quality of each piece of evidence will be graded into four levels based on the GRADE rating scale: very low, low, moderate and high.

### Ethics and data privacy considerations

There is no need to be concerned about the privacy of patient data for this study, as the data used will come from previously published trials. This review will not involve any human participants and does not require ethical approval.

## Discussion

Compared to face-to-face healthcare, mobile apps are easier to reach patients, overcome time and space limitations, and can be integrated into their daily lives, making them more practical [20]. Through the introduction of game items, the entertainment element of mobile apps can also assist patients in developing their coping skills with health issues and enhance treatment adherence [21]. They might therefore be more skilled at using self-management to raise their standard of living. In addition, as the high economic burden of cancer can further exacerbate the physical health of cancer patients, mobile applications without direct contact with hospitals or clinic service organizations may have a certain cost-reduction effect.

To further improve the cancer patients’ quality of life, it is vital to scientifically and methodically assess the profound impact of mobile applications. This will not only validate the application of scientific and technological advances in medicine, but also provide strong evidence-based medical support to healthcare professionals, allowing them to better use these tools to develop more personalized and efficient patient treatment and care. To be specific, determining whether apps with specific features can considerably benefit QoL in cancer patients will assist clinicians in developing personalized intervention plans according to patients’ specific needs, disease types, and stages, optimize existing treatment strategies, incorporate apps into treatment plans as an auxiliary treatment method, enhance treatment efficacy, and improve patients’ self-management ability. At the same time, the convenience and ease of use of apps will enable patients to take a more active role in their health management, support physicians in proposing relevant apps to patients, and enhance treatment compliance and QoL. In terms of quality of life, app interventions have been demonstrated to have an influence on multiple dimensions such as physical, role, emotional, cognitive and social functions, allowing patients to better cope with disease-related challenges, maintain a positive attitude, and reintegrate into society.

There are numerous important possible challenges in moving forward with this study. The first is the large quantity of articles, as well as the variety of disease types and research methodologies, which considerably complicate article classification and may result in incorrect classification, reducing the study’s overall power and accuracy. We will create and apply more severe and detailed screening criteria. This can efficiently minimize the number of publications while ensuring that the selected literature is highly representative and scholarly, thus laying the groundwork for further study. Second, there could be significant heterogeneity due to differences in sample size, intervention content, timing, frequency, and evaluation instruments, and we understand how important it is to deal with and explain heterogeneity in order to ensure the reliability and robustness of research conclusions. As a result, in the study report, we will go over the heterogeneity treatment approach, as well as thoroughly explore the potential impact of heterogeneity on the research conclusions. Third, this study is confined to analyzing published publications and only contains English or Chinese literature, neglecting relevant data in other languages, which may cause publication bias and language bias. Fourth, this study may not include data from all countries and areas. Given the variable impact of different cultural environments on the study results, the characteristics of multiculturalism symbolize a potential limitation of this study, implying that the general applicability of the conclusions in different cultural backgrounds needs further verification. We expect that by using such rigorous study design and analytic methods, we will be able to provide researchers in this subject with more scientific and credible research data while also promoting the ongoing development and progress of related fields.

We also made a preliminary estimate of the obstacles that might be encountered during the study. Firstly, the wide variety of mobile apps with different functions may have varying effects on the quality of life of cancer patients. Therefore, these interventions need to be carefully screened and categorized to ensure the validity of the analysis. If heterogeneity is discovered, we will utilize graphical approaches (Galbraith chart, L’Abbe chart) or statistical methods (Q test, I^2^ test) to test it, explain its impact on the results, and suggest future research areas. Secondly, not all studies make their data publicly available or store them in easily accessible databases; if data are not available, the researchers could be contacted directly. Third, because quality of life is a complex concept, different studies may evaluate it using dissimilar definitions and measurement tools, making direct comparisons and combinations of data hazardous. This can lead to inconsistencies in the results. Therefore, the reliability of the results could be enhanced through a clear definition of quality of life, tools to measure quality of life, careful screening and integration of data.

## Conclusion

We will draw conclusions based on the outcomes of the study.

## Supporting information

S1 Table The pre-defined electronic form to extract the characteristics of the included RCTs. (DOCX)

S2 Table Checklist. PRISMA-P 2015 checklist.
(DOCX)
