# Supplementary material for: Effects of mobile application interventions on quality of life in patients with cancer: Protocol for a systematic review and meta-analysis
Source: PLoS One. 2025 Feb 12;20(2):e0314590. doi: 10.1371/journal.pone.0314590 (PMC11819588; doi:10.1371/journal.pone.0314590)
Supplement: S1 Table — (DOCX) [file pone.0314590.s001.docx]

**Supplementary Table 1. The pre-defined electronic form to extract the characteristics of the included RCTs.**

| **Author,**  **Year,**  **Country** | **Sample size** | | **Patient characteristics** | | | | | **Intervention** | **Control** | **Duration of intervention** | **Outcome measurement** |
| --- | --- | --- | --- | --- | --- | --- | --- | --- | --- | --- | --- |
|  | **Intervention** | **Control** | **Mean age (years), intervention** | **Mean age(years), control** | **Type of cancer** | **Stage of cancer** | **treatment strategy** |  |  |  |  |
| Paper 1 et al |  |  |  |  |  |  |  |  |  |  |  |
| Paper 2 et al |  |  |  |  |  |  |  |  |  |  |  |
| Paper 3 et al |  |  |  |  |  |  |  |  |  |  |  |
| Paper 4 et al |  |  |  |  |  |  |  |  |  |  |  |
| Paper 5 et al |  |  |  |  |  |  |  |  |  |  |  |
| Paper 6 et al |  |  |  |  |  |  |  |  |  |  |  |
| Paper 7 et al |  |  |  |  |  |  |  |  |  |  |  |
| Paper 8 et al |  |  |  |  |  |  |  |  |  |  |  |
| Paper 9 et al |  |  |  |  |  |  |  |  |  |  |  |
| Paper 10 et al |  |  |  |  |  |  |  |  |  |  |  |
| Paper 11 et al |  |  |  |  |  |  |  |  |  |  |  |
| Paper 12 et al |  |  |  |  |  |  |  |  |  |  |  |
| Paper 13 et al |  |  |  |  |  |  |  |  |  |  |  |
| Paper 15 et al |  |  |  |  |  |  |  |  |  |  |  |
| Paper 16 et al |  |  |  |  |  |  |  |  |  |  |  |
| Paper 17 et al |  |  |  |  |  |  |  |  |  |  |  |
| Paper 18 et al |  |  |  |  |  |  |  |  |  |  |  |
| Paper 19 et al |  |  |  |  |  |  |  |  |  |  |  |
| Paper 20 et al |  |  |  |  |  |  |  |  |  |  |  |
